# Effects of a guided digital intervention on sleep and mental health outcomes in university students – a randomized controlled trial

**DOI:** 10.1093/sleep/zsaf357

**Published:** 2025-11-25

**Authors:** Laura Michelle Pape, Annemieke van Straten, Sascha Yuri Struijs, Julian David Karch, Philip Spinhoven, Niki Antypa, Caring Universities Consortium

**Affiliations:** Department of Clinical Psychology, Leiden University, Leiden, the Netherlands; Department of Clinical, Neuro- and Developmental Psychology, VU University Amsterdam, Amsterdam, the Netherlands; Amsterdam Public Health Research Institute, Vrije Universiteit Amsterdam, Amsterdam, the Netherlands; Department of Clinical, Neuro- and Developmental Psychology, VU University Amsterdam, Amsterdam, the Netherlands; Department of Methodology and Statistics, Leiden University, Leiden, the Netherlands; Department of Clinical Psychology, Leiden University, Leiden, the Netherlands; Department of Clinical Psychology, Leiden University, Leiden, the Netherlands

**Keywords:** E-health, cognitive behavioural therapy, insomnia, circadian rhythm, university, students

## Abstract

**Study Objectives:**

To evaluate the effectiveness of a guided digital intervention targeting sleep and circadian rhythms in university students.

**Methods:**

We randomized 195 university students with self-reported insomnia (Insomnia Severity Index ≥10) from nine Dutch Universities to receive either the 5-week guided digital cognitive-behavioral therapy for insomnia *“i-Sleep & BioClock”,* or digital sleep psychoeducation and sleep monitoring. Online assessments were at baseline, 3 (mid-treatment), 6 (post-treatment), and 18 (follow-up) weeks. The primary outcome was insomnia severity at 6 weeks. Secondary outcomes were symptoms of depression and anxiety; functioning; quality of life (QoL); academic performance; and sleep and light exposure diary outcomes.

**Results:**

From Nov 1^st^ 2023 until Sept 5^th^ 2024, we randomly assigned 96 students (age 23.8 ± 3.8, 75.4% female) to the intervention and 99 to the control condition, stratified by university and gender. Fifty-one percent completed the post-treatment assessment, and 30% the follow-up. 31% were intervention completers. Linear mixed models showed no significant time x treatment interaction for insomnia severity (*“i-Sleep & BioClock”*: baseline ISI M = 16.8, SD = 4.0; post-treatment ISI M = 10.2, SD = 3.9; Digital psychoeducation: baseline ISI M = 16.5, SD = 3.6; post-treatment ISI M = 11.1, SD = 4.2). The difference in change between groups was -1.17 points (95% CI –2.72 to 0.38), *p*=.14, Cohen’s *d* = -0.31. Most secondary outcomes showed no significant time x treatment effects.

**Conclusions:**

*“i-Sleep & BioClock”* was not more effective than online psychoeducation on most outcomes, possibly due to active elements present in both conditions or low adherence. Future research should identify factors for early disengagement and investigate which intervention components drive improvements in sleep.

**Clinical Trial:**

Effectiveness of a Guided Self-help Intervention for Improving Sleep in University Students, https://clinicaltrials.gov/study/NCT06023693, prospectively registered at ClinicalTrials.gov on August 3^rd^, 2023.

## Teaser

**Statement of Significance** Sleep problems are highly prevalent among university students and are associated with poor mental health, functioning, and academic outcomes. Students struggle to maintain a 24-hour rhythm which is why we enhanced a digital CBT-I intervention with elements targeting the circadian rhythm. Although there is considerable evidence to support the effectiveness of CBT-I in various populations, this study shows that digital CBT-I is not superior to digital psychoeducation and sleep monitoring in students. Both groups showed notable improvements, likely reflecting active elements in both conditions. Low adherence may also have diminished the between-group effects. Future research should explore the potential of brief digital sleep interventions, barriers to adherence, and investigate which intervention components drive improvements in sleep.

## Graphical Abstract

**Graphical Abstract:**
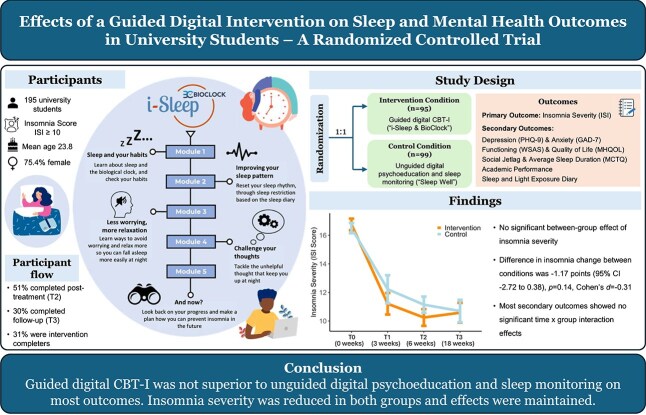

## Introduction

Insomnia is a major public health problem. Insomnia disorder is characterized by night-time symptoms including difficulties falling asleep or maintaining sleep, and daytime symptoms such as fatigue, lack of concentration, and mood problems, leading to impairment in functioning [1]. Insomnia is very common in university students, with prevalence numbers ranging from 10-30% [2–5]; the prevalence of sleep problems in general (also including e.g., circadian rhythm disorders or parasomnia) in this population reaches up to 60% [5, 6]. It is well known that insomnia has detrimental consequences on physical health [7, 8], mental health [9, 10], functioning, and performance [11, 12]. Insomnia is also recognized as a transdiagnostic risk factor for the development of several mental disorders, such as depression, anxiety, psychotic disorders, and suicidal ideation [7, 13]. A recent meta-analysis found that individuals with insomnia have 2.74 higher odds (95% CI 1.82 to 4.12) of developing a mental disorder, particularly a depressive disorder [14]. It has also been demonstrated (in elderly people) that treating insomnia can actually delay or prevent the onset of depression [15]. Improving sleep in young people might therefore be important to prevent the development of mental disorders. Taken together, the high prevalence and impact of sleep problems in students warrant the need to improve and evaluate existing treatments.

The established first-line treatment for sleep problems, according to clinical guidelines (e.g., European guidelines) [16], is cognitive-behavioral therapy for insomnia (CBT-I). CBT-I is also available in digital format, which offers many advantages, and is a viable alternative for face-to-face CBT-I [17]. CBT-I often shows large effects on insomnia and also moderate effects on mental health related outcomes such as depression and anxiety [18–20]. In students and young adults, the effects of CBT-I seem to be somewhat smaller than in the general population, yet clinically relevant, with moderate to large effects in sleep outcomes (*d* = 0.5 to 0.8) and small to moderate effects in mental health outcomes (*d* = 0.2 to 0.5) [21–23]. Although the establishment of a 24-hour rhythm is an important element in insomnia treatment, existing CBT-I protocols seldom include specific information and exercises related to the circadian rhythm.

The circadian rhythm is the body’s internal clock which regulates many physiological and behavioural processes across a 24-hour cycle. It is especially important for university students, whose schedules and demands make them prone to having irregular rhythms [24]: irregular sleep and wake times, irregular meals, and improper timing of exposure to light. Irregular rhythms can lead to circadian misalignment, since the circadian system is influenced by external stimuli (also known as “*zeitgebers*”), such as light and physical activity [24, 25]. A common form of circadian misalignment is social jetlag, a discrepancy of sleep/wake schedules between work/study days and free days [26, 27]. A social jetlag of one hour per week is present in at least 70% of students and working population [28]. Social jetlag is associated with adverse health behaviors like smoking and alcohol consumption and has furthermore been associated with lower academic performance, metabolic and cardiovascular diseases, and adverse mental health [26]. Knowledge and awareness about the body’s circadian system, and concrete tips to improve the biological clock, might enhance the effectiveness of CBT-I. Earlier studies have shown that for patients with insomnia and high risk of depression, adding circadian rhythm support to CBT-I leads to better long-term effectiveness in sleep outcomes and depressive symptoms [29].

In this study we used the existing digital CBT-I intervention *“i-Sleep”* and adapted it specifically for students by placing special emphasis on the circadian rhythm. We tested the effectiveness of this new intervention (*“i-Sleep & BioClock”*), compared to digital psychoeducation and sleep monitoring, in a randomized controlled trial for university students with self-reported insomnia. The primary aim of this study was to evaluate the effects of the intervention in reducing insomnia severity. The secondary aims were to examine the effects on other sleep and circadian rhythm-related outcomes, depression and anxiety severity, functioning and quality of life, and academic performance. Moreover, we investigated whether effects were maintained at 18-week follow-up. We hypothesized that the guided CBT-I intervention would lead to significantly better sleep and mental health outcomes than the digital psychoeducation and sleep monitoring condition, and that effects would be maintained.

## Materials and Methods

### Study design and setting

This study was a two-arm parallel group randomized controlled trial comparing a guided digital CBT-I intervention versus digital psychoeducation and sleep monitoring. The study was conducted under pragmatic conditions in the routine university setting. All procedures such as screening, informed consent, assessments, allocation to conditions, and interventions were carried out via an automated online platform (*“MoodLift”* [30]). The trial received ethical approval by the METC Leiden Den Haag Delft on July 17^th^, 2023 (NL83395.058.23) and approval by the local ethical committee at Leiden University (Psychology Research Ethics Committee) on October 23^rd^, 2023. The trial was registered at ClinicalTrials.gov (NCT06023693), and we reported it according to the CONSORT guidelines for randomized controlled trials [31].

### Participants

Recruitment started on November 1^st^, 2023 and ended when we reached our target sample size on September 5^th^, 2024. Recruitment methods included the annual Caring Universities mental health screening survey, on-site advertisements at the universities, social media postings, and referrals of university staff including study advisors or student psychologists. We included university students aged ≥16 years who were enrolled as Bachelor, Master, or PhD students at one of nine Dutch universities connected to the Caring Universities Consortium. Students were eligible if they were able to speak Dutch or English and if they had self-reported sleep problems, as indicated with a score of ≥10 points on the Insomnia Severity Index (ISI). Students were excluded if they had a current risk for suicidal behavior (as indicated by reporting suicidal thoughts in the past 12 months and not ruling out the possibility to act on these thoughts in the next year) and if they reported regular night shifts (meaning work between 2 a.m. and 6 a.m. at least once a week). For more details see the study protocol [32].

### Randomization and blinding

We used 1:1 block randomization via an automated online system built in with the *“MoodLift”* platform, stratified by gender and university. Conditions were automatically assigned upon completion of the baseline assessment. This automated procedure ensured that the allocation was blinded. The study was an open label trial in which nobody was blinded. The website of the control intervention was clearly labeled as *“Sleep Well”*, differentiating it from the *“i-Sleep & BioClock”* intervention.

### Intervention – Guided digital CBT-I

Participants in the intervention condition received the *“i-Sleep & BioClock”* intervention, which is a 5-week digital guided intervention. The intervention was provided in five modules, each of which take 30 to 60 minutes to read. Based on this information the participants had to implement behavior changes (e.g., restricting hours in bed, creating a bedtime ritual). The intervention was based on cognitive behavioral therapy for insomnia (CBT-I), such as guided sleep monitoring using the techniques of keeping a sleep and light exposure diary, sleep restriction, cognitive restructuring, and relaxation techniques. These traditional CBT-I elements were enriched with elements targeting the circadian rhythm, such as information on the importance of timed light exposure, social jetlag, and chronotype. The intervention and the sleep and light exposure diary were accessible via the browser or smartphone. Students were advised to monitor their sleep with the sleep and light exposure diary, which was evaluated each week. The intervention was guided by online coaches, who provided an intake call as well as textual asynchronous feedback to the participants in form of a chat on the platform, reacting to the written exercises and progress reports. The online coaches were third-year bachelor students of clinical psychology who were trained with two workshops and supervised with regular face-to-face meetings by a licensed psychologist. The intervention protocol is described in the study protocol [32] and summarized in Table 1.

**Table 1:** Content of the *“i-Sleep & BioClock”* intervention

**#**	**Module**	**Content**
**1**	**Sleep and your habits**	**Psychoeducation about sleep and the circadian rhythm** (Two Process Model of Sleep, Sleep stages, Sleep as a 24-hour phenomenon, role of the circadian rhythm, chronotypes). **Chronotype questionnaire** and feedback. **Assessment of own lifestyle and habits** (bedroom, evening ritual, light exposure, alcohol, caffeine, smoking) and goal setting to change some of these habits. **Sleep monitoring with the sleep and light exposure diary**.
**2**	**Changing your sleep pattern**	**Psychoeducation on insomnia** (3-P Model of Insomnia, Downward Spiral, Consequences of Insomnia, Education on Sleep Medication), Psychoeducation on **social jetlag** and **importance of regularity** and establishing a 24-hour rhythm. Sleep inertia and tips to get up better in the morning. **Sleep Restriction** and **Stimulus Control** and goal setting. Case examples of other students.
**3**	**Less worrying, more relaxation**	**Practical exercises** to reduce **worrying** and increase **relaxation**. Regular evaluation of thoughts and worries. Trying to stay awake exercise, Blocking your thoughts exercise and Progressive Muscle Relaxation Exercises.
**4**	**Changing your thoughts**	Common misconceptions and **dysfunctional thoughts and beliefs about sleep**. **Cognitive restructuring** (Identifying own dysfunctional thoughts and replacing them with more adaptive sleep-promoting thoughts).
**5**	**And now?**	**Summary** of the intervention content. **Process evaluation** (review of the sleep and light exposure diary and treatment progress). **Relapse prevention**.

### Control group – Digital psychoeducation and sleep monitoring

Participants in the control condition received a brief online psychoeducation program (*“Sleep Well”*), consisting of seven very brief lessons that each took on average 5 to 10 minutes to complete. Although these were divided as separate lessons, the content was very condensed and comparable to Module 1 (psychoeducation) of the *“i-Sleep & BioClock”* intervention. The control condition included basic information about sleep and circadian rhythms (see Table 2), as well as access to a sleep and light exposure diary. This condition was not coach-assisted.

**Table 2:** Content of the *'Sleep Well'* psychoeducation

**#**	**Lesson**	**Content**
**1**	**Introduction**	Introduction to the program. Sleep monitoring with the sleep and light exposure diary.
**2**	**Why do we sleep?**	Sleep for physical and mental well-being.
**3**	**How does sleep work?**	Psychoeducation about sleep (Two Process Model of Sleep, sleep stages).
**4**	**Sleep as a 24-hour phenomenon**	Basic sleep hygiene (the bedroom, evening ritual, light exposure, general lifestyle advice) and Stimulus Control.
**5**	**Sleep and the downward spiral**	Psychoeducation on insomnia (3-P Model of Insomnia and the Downward Spiral).
**6**	**The biological clock**	Psychoeducation on the circadian rhythm (role of the circadian rhythm, chronotypes, social jetlag). Chronotype assessment and automated feedback.
**7**	**Summary**	Summary of the sleep hygiene advice.

Compared to the intervention, the control condition contained no guidance, substantially minimized and plain content, and did not include key behavioral components such as sleep restriction. Additionally, the control condition lacked typical cognitive techniques such as cognitive restructuring, as well as relaxation exercises, which were integral parts of the intervention. After the study concluded, students in the control group were offered the intervention, however, only three students chose to take the offer.

### Measurements

Assessment points were at baseline and 3 weeks (T1), 6 weeks (T2), and 18 weeks (T3) after baseline. T2 was the primary endpoint. The week 3 assessment (T1) comprised only the primary outcome measure and measures used for mediation analysis. Students received €15 for completing T2 and were entered into a raffle for 8 prizes of €50 upon completing T3.

#### Primary outcome

The primary outcome was *insomnia severity* measured with the 7-item Insomnia Severity Index [ISI] [33]. Total scores range from 0-28 with higher score indicating higher insomnia severity. A reduction in the total ISI score of >8 was defined as clinically significant response [33].

#### Secondary outcomes

##### Sleep and Light Exposure Diary Outcomes

Secondary sleep outcomes were assessed with the sleep and light exposure diary. We asked participants to fill in the diary each day in the morning, in the first seven days after baseline measurement (week 1) and in the seven days after the post-test measurement (week 7). Diary entries during the whole period were encouraged. We measured *sleep onset latency* [SOL], *wake after sleep onset* [WASO], *early morning awakening* [EMA], *total sleep time* [TST], *sleep efficiency* [SE], *sleep quality*, and *feeling rested in the morning*. The diary furthermore assessed *light exposure* and *screen use before bedtime*. Details on the items can be found in our study protocol [32].

##### Chronobiology Outcomes

*Average weekly sleep duration* (in minutes) and *social jetlag* (the difference in mid-sleep between work/study days and free days) were derived from the Munich Chronotype Questionnaire [34] (MCTQ). The MCTQ assesses bedtime and wake time on work/study days and on free days over the past four weeks, based on which the outcomes are calculated.

##### Mental Health and Functioning Outcomes

We assessed *depression* (9-item Patient-Health-Questionnaire [35] [PHQ-9] using the full scale), *anxiety* (7-item Generalized-Anxiety-Disorder Scale [36] [GAD-7]), *impairment in functioning* (Work and Social Adjustment Scale [37] [WSAS]), and *quality of life* (Mental Health Quality of Life Questionnaire [38] [MHQoL]). Higher scores on PHQ-9 (range 0-27), GAD-7 (range 0-21) and WSAS (range 0-40) reflect worse mental health symptoms and functioning, respectively. Higher scores on MHQoL (range 0-21) reflect better QoL. For depressive symptoms, a reduction of 5 points on the PHQ-9 was considered the minimal clinically important difference (MCID) [39]. For anxiety symptoms, the MCID on the GAD-7 total score was a reduction of 4 points [40]. Participants received automated feedback on the baseline scores of ISI, GAD-7 and PHQ-9, advising to seek additional professional help (e.g., student psychologist or GP) in case of moderate to severe anxiety or depressive symptoms. Furthermore, we assessed *academic performance* with multiple indicators: average GPA of past semester [1–10], last exam grade (1–10), failed courses in the past semester (Yes/No), and study progression (30 ECTS per semester: Yes/No).

#### Other outcomes

We used the Client Satisfaction Questionnaire (CSQ-8) to assess *user satisfaction* with the intervention and the online psychoeducation (score range 8-32) [41]. Higher scores refer to higher user satisfaction.

In the intervention group, we assessed *therapeutic alliance* with the e-coaches, which was measured with the Working Alliance for guided Internet Intervention Scale (WAI-I) with a score range of 12-60 [42]. Higher scores reflect better therapeutic alliance.

For *adherence*, we used several measures such as the number of logins and time spent on the platform. The main measure for adherence were the number of modules completed in the intervention group and number of lessons completed in the control group, based on which we calculate the module/lesson-based adherence (the percentage of total number of modules/lessons completed in each condition divided by the total amount of possible modules/lessons). We defined intervention completers as ≥4 *“i-Sleep & BioClock”* modules completed or ≥ 6 *“Sleep Well”* lessons. Adverse events were measured at T1 and T2. More details on the various measures and timing of measurements can be found in the study protocol [32].

### Sample size

We calculated the required sample size calculation to detect a small-to-medium effect (Cohen’s *d* = 0.4) on the primary outcome, insomnia severity, which is slightly more conservative compared to the effect sizes reported in prior literature [21]. Assuming a 80% power, a two-sided significance level of *α* = 0.05, and taking into account the repeated measures design with four time points and an estimated intraclass correlation (ICC) of 0.23, we estimated that 48 participants per condition were needed. To account for an anticipated 50% dropout rate, as observed in similar digital intervention trials [18], we aimed to enroll 192 participants in total [43].

### Statistical analysis

All analyses were performed in R (version 4.2.1). Between-group differences at baselines were tested with independent samples t-tests for continuous variables and Fisher’s exact tests for categorical variables to assess whether randomization was successful.

Data were analyzed by intention-to-treat principle. No data imputation was performed, instead, missing values were handled using linear mixed models (LMM), which use all available observations under the missing at random assumption. To analyze the primary and secondary outcomes, we conducted separate LMMs including the outcome variable, time (T0, T1, T2, and T3) and group (intervention versus control) with random intercepts. Random slopes for time were added if the AIC indicated an improvement of the model. All models were checked for linearity between fixed effects and outcome and homoscedasticity of residuals by plotting raw, Pearson and standardized residuals against fitted values. The distribution of random effects was checked with QQ-plots. Normality of the residuals was evaluated visually through QQ-plots and histograms overlaid with a normal density curve. If assumptions were violated, we used bootstrapping with 1000 semiparametric resamples to compute robust 95% confidence intervals for fixed effects. In case the results were similar to the original LMM, we reported the estimates of the original model. We used estimated marginal means (least-squares means) from the model and computed difference-in-differences contrasts to test whether changes in ISI scores from baseline to T2 and T3 differed between the two conditions. We calculated the within-group and between-group effect sizes (standardized difference in change between intervention and control) with Cohen’s *d_p_*, in which the difference between two means is divided by the pooled standard deviation of the groups or time points [44]. According to convention, this can be interpreted as small (~0.2), medium (~0.5), or large (>0.8) [44]. To account for false positives due to multiple hypothesis testing, we applied Holm’s correction to the *p*-values for the eight main interaction effects [45].

We conducted a sensitivity analysis for the primary outcome (ISI) to assess the potential impact of missing data on our results by conducting a pattern-mixture model [46]. This was a linear mixed model including an interaction with a missingness indicator for the ISI at T2, which was compared to the original LMM in terms of model fit. Estimated marginal means were computed separately for participants with and without missing data at T2 to compare trajectories of insomnia severity over time.

Sleep and light exposure diary outcomes were analyzed using LMM for the approximately continuous variables (TST, SE, light exposure, sleep quality, and feeling refreshed in the morning) and negative binominal mixed models (NBMM) for count variables (SOL, WASO, EMA). Random intercepts and slopes or random intercepts only were considered, and models were compared with likelihood ratio tests. Power transformation was applied to the negatively skewed SE variable (SE/100*3), and square root transformation was used for the skewed light exposure variable before applying the LMM. SOL, WASO, and EMA were recoded to represent counts (1 count per 15 minutes). A continuous autocorrelation structure of order 1 was used to account for missing data and unequally spaced observations for the regular LMMs. Logistic binominal regression using a generalized linear mixed model (GLMM) was performed for the binary outcome screen use before bedtime. For the GLMM and NBMM models a continuous autocorrelation structure could not be included because it was not available in the respective R packages (*lme4*, *glmmTMB*).

## Results

### Response

**Figure 1:**
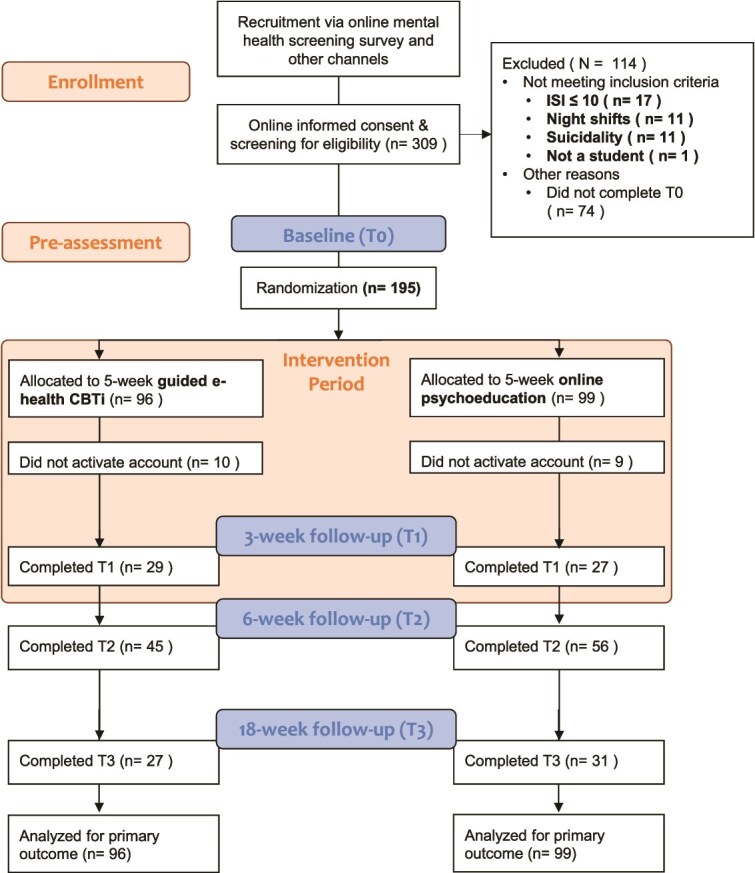
CONSORT flow diagram.

In total, 309 students registered for the intervention between November 2024 and September 2025 and provided informed consent. Of the 309 registered students, 114 (36.9%) did not meet the inclusion criteria. Eleven students were excluded because of suicidal ideation (11/309, 3.5%), 17 (17/309, 5.5%) because they had an ISI score below 10, 11 (11/309, 3.5%) because of regular night-shifts, one was not a student, and 74 (74/309, 23.9%) because they did not complete the baseline assessment (Fig. 1). A total of 195 students were included and randomized to the intervention group (*n* = 96) or the active control group (*n* = 99). In total, 195 students (100%) completed the T0 assessment, 56 (29%) completed the T1 assessment (mid-treatment), 101 (52%) completed the T2 assessment (end of treatment), and 58 (30%) completed the T3 assessment (three months after the end of treatment), see Fig. 1. The number of responses to study assessments at all assessment points was not significantly different between the two groups.

Of the 195 included participants, 116 filled in the sleep and light exposure diary at least once: 54 students (56%) in the intervention group and 62 students (62%) in the control group. The total number of entries in the intervention group was 1264, with a mean of 23.4 (SD 19.1) per participant, and the total in the control group was 645 entries, with a mean of 10.4 (SD 13.1) per participant.

### Sample Descriptives

The RCT included 195 university students. The mean age was 23.8 years (SD 5.2), and 75.4% were female. The mean baseline score of insomnia severity was 16.6 (SD 3.8), meaning moderate insomnia severity [33]. There were no significant differences in baseline characteristics between the two groups. The majority of our participants (87%) had never used an online mental health program before. Most of them (77%) did not currently receive any medication or therapy (Table 3). Among the 45 students receiving treatment, 14 participants (31%) reported more than one type of therapy or medication. A total of 26 participants (57% of those receiving treatment) reported engaging in psychotherapy. The most commonly reported approach was cognitive behavioral therapy (CBT), reported by 8 participants (31% of those in psychotherapy). Other approaches included schema therapy (*n* = 3), EMDR (*n* = 2), psychodynamic therapy (*n* = 1), and acceptance and commitment therapy (*n* = 1), and other/unspecified approaches (*n* = 11). Thirty participants (67% of those receiving treatment) reported using medication. The most reported types of medication were medication for ADHD (*n* = 12, 40%), such as methylphenidate, and antidepressants (*n* = 12, 40%), such as selective serotonin reuptake inhibitors. Additional medications included sleep aids such as melatonin, trazodone (*n* = 6), anxiolytics such as oxazepam, clonazepam (*n* = 3), and mood stabilizers such as lamotrigine (*n* = 1).

**Table 3:** Baseline demographic characteristics of all included participants (*n* = 195)

**Demographic characteristics**		**Intervention (n = 96)**	**Control (n = 99)**
**Age, years, mean (SD)**		23.4 (4.3)	24.1 (6.0)
**Gender, female, n (%)**		74 (77.1)	73 (73.7)
**University, n (%)**			
	University of Amsterdam	16 (16.7)	15 (15.2)
	Leiden University	27 (28.1)	25 (25.3)
	Vrije Universiteit Amsterdam	5 (5.2)	7 (7.1)
	Erasmus University Rotterdam	16 (16.7)	16 (16.2)
	Maastricht University	9 (9.4)	10 (10.1)
	Utrecht University	13 (13.5)	14 (14.1)
	Hogeschool Rotterdam	9 (9.4)	9 (9.1)
	Inholland Hogeschool	1 (1.0)	1 (1.0)
	Avans Hogeschool	0 (0.0)	2 (2.0)
**Level of education, n (%)**			
	First-year student	18 (18.8)	22 (22.2)
	Second-year student	7 (7.3)	10 (10.1)
	Third-year student	15 (15.6)	19 (19.2)
	Fourth-year student	13 (13.5)	9 (9.1)
	Master student	40 (41.7)	37 (37.4)
	PhD student	3 (3.1)	2 (2.0)
**Nationality, n (%)**			
	European (Dutch)	55 (57.3)	58 (58.6)
	European (not Dutch)	32 (33.3)	25 (25.3)
	Non-European	9 (9.4)	15 (16.1)
**Relationship status, n (%)**			
	Single	50 (52.1)	54 (54.5)
	Other	44 (47.9)	45 (45.5)
**Children, n (%) yes**		1 (0.01)	1 (0.01)
**Living Situation, n (%)**			
	On my own	31 (32.3)	24 (24.2)
	With parents	17 (17.7)	22 (22.2)
	With partner	9 (9.38)	12 (12.2)
	With flat mates	39 (40.6)	41 (41.4)
**Part-time job, n (%) yes**		55 (57.3)	53 (53.5)
**Duration of sleep problems, years**			
	< 1 year	34 (35.4)	42 (42.4)
	1 - 4 years	31 (32.3)	25 (25.3)
	> 4 years	31 (32.3)	32 (32.3)
**Use of medication or psychotherapy, n (%)**			
	Medication	7 (7.3)	12 (12.1)
	Psychotherapy	6 (6.3)	10 (10.1)
	Both	3 (3.1)	7 (7.1)
	None	80 (83.3)	70 (70.7)
**Alcohol consumption**			
	Drinker, n (%)	73 (76.0)	73 (73.7)
	Drinks/week, n (SD)	6.0 (7.7)	6.2 (7.7)
**Smoking**			
	Smoker, n (%)	23 (24.0)	17 (17.2)
	Cigarettes/week, n (SD)	10.6 (17.8)	22.1 (40.2)
**Baseline symptoms, mean (SD)**			
	Insomnia Severity (ISI)	16.8 (4.0)	16.5 (3.6)
	Depression (PHQ-9)	11.7 (4.0)	11.1 (4.8)
	Anxiety (GAD-7)	9.7 (4.7)	8.8 (5.0)
	Functioning (WSAS)	20.7 (6.9)	20.6 (8.7)
	Quality of Life (MHQoL)	12.4 (2.8)	12.6 (3.1)

### Intervention uptake and engagement

In terms of uptake, after randomization, 21 students (19/195, 9.7%) did not activate their user account (see Fig. 1). Out of the 176 with an activated account, 26 students never logged into the platform (15%), with no significant difference between groups. Table 4 shows an overview of completed modules per condition. Out of the 150 intervention initiators, 31% of intervention group participants (23/74) completed the intervention (4 or more modules). In the control group, 61% of intervention initiators (46/76) completed the intervention (6 or more lessons). The module completion rate in the intervention group was 42.7%, derived from total number of modules (*n* = 158) completed by intervention initiators out of the possible number of modules *n* = 370 (74*5). In the control group, the lesson completion rate was 68.4% based on the total number of lessons (*n* = 364) divided by the possible number of lessons *n* = 532 (76*7). Although completion appears higher in the control group, the lessons were considerably shorter than the modules, which is reflected in overall engagement: those in the intervention condition logged in more often than those in the control condition (M = 26, SD = 28.2 vs. M = 12, SD = 14.2) and spent more time on the platform (M = 104, SD = 112.0 minutes vs. M = 26, SD = 23.8 minutes). The most mentioned reasons for discontinuation were lack of time (*n* = 9) and loss of interest/motivation (*n* = 7); full details are provided in Table S1 of the Supplementary Material.

**Table 4:** Completion of Modules (Intervention) versus Lessons (Control)

**Intervention group (n = 96)**		**Control group (n = 99)**	
Module	n completed	Lesson	n completed
Module 1	49 (51%)	Lesson 1	66 (67%)
Module 2	37 (39%)	Lesson 2	59 (60%)
Module 3	28 (29%)	Lesson 3	54 (55%)
Module 4	23 (24%)	Lesson 4	48 (48%)
Module 5	21 (22%)	Lesson 5	46 (46%)
-	-	Lesson 6	46 (46%)
-	-	Lesson 7	45 (45%)

*Note.* Intervention comprised of 5 modules, control group comprised of 7 very brief lessons.

### Primary outcome

**Figure 2:**
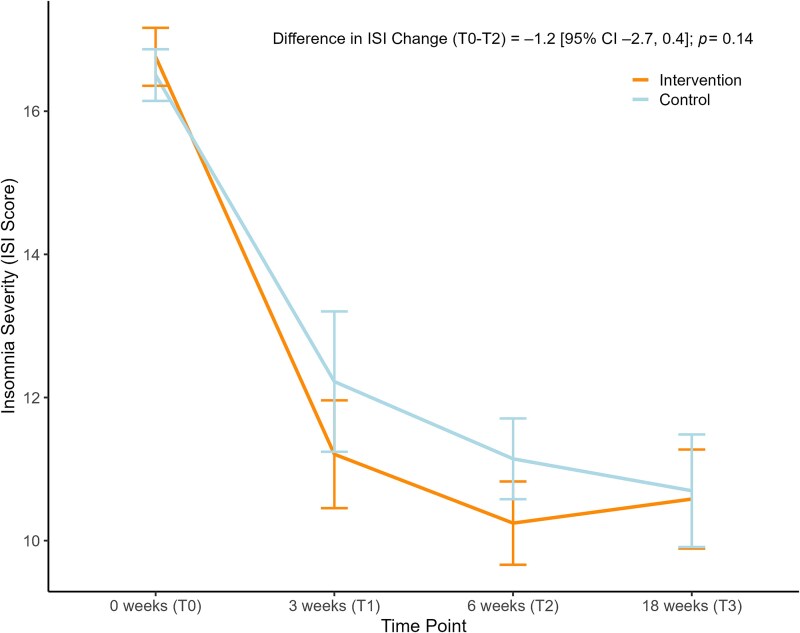
Insomnia severity index scores on all time points for intervention and control group. *Note.* Changes in mean insomnia severity over time split by group. Values are observed means of the primary outcome. Error bars represent standard errors. T0 = baseline, T1 = 3 weeks mid-treatment, T2 = 6 weeks post-treatment, T3 = 18 weeks follow-up.

Insomnia severity was significantly reduced in both groups, with a large within-group Cohen’s *d* of -1.72 in the intervention group and *d* = -1.42 in the control group at 6 weeks. Figure 2 displays the group means and standard deviations of ISI at all time points. As indicated by the non-significant time x treatment interaction, the *i-Sleep & BioClock* intervention did not have superior effects on insomnia severity compared to online psychoeducation, at 6 weeks *t*(279.6) = -1.49, *p*=.13, nor at 18 weeks *t*(284.5) = -0.52, *p*=.60. The difference in change between groups from T0 to T2 was -1.17 points (95% CI –2.72 to 0.38), *p*=.14, indicating a non-significant difference in change between the two conditions from baseline to primary endpoint. The effect size for the difference in change of ISI from T0 to T2 (Cohen’s d) was *d* = -0.31.

The pattern mixture model (sensitivity analysis) showed no significant difference in model fit compared to the original model, *χ^2^*(6) = 1.93, *p*=.93, and all interaction with the missingness indicator were non-significant. Trajectories of insomnia severity were similar for participants with and without missing data at the primary endpoint, indicating robustness of the results.

Regarding follow-up, the difference in change between T0 to T3 between the conditions was –0.49 points (95% CI –2.33 to 1.36), *p*=.60, indicating no significant difference in change form baseline to follow-up. The effect size for the difference in change of ISI from T0 to T3 was *d* = -0.10. Means, standard deviations, and effect sizes of primary and secondary outcomes can be found in the Supplementary Material Table S2 and estimates of linear mixed models are shown in Table S3 (Supplementary Material).

### Clinically meaningful insomnia change

In the 101 students who completed the T2 assessment, clinically significant treatment response at T2, defined as reduction in total ISI score of ≥8 points, occurred in 42% (*n* = 19/45) students in the intervention group and in 27% (*n* = 15/56) students in the control group. The difference between groups was not statistically significant, *χ^2^*(1) = 2.02, *p=*.16. None of the students experienced clinically significant worsening of insomnia severity.

### Secondary outcomes

#### Sleep and light exposure diary outcomes

**Figure 3:**
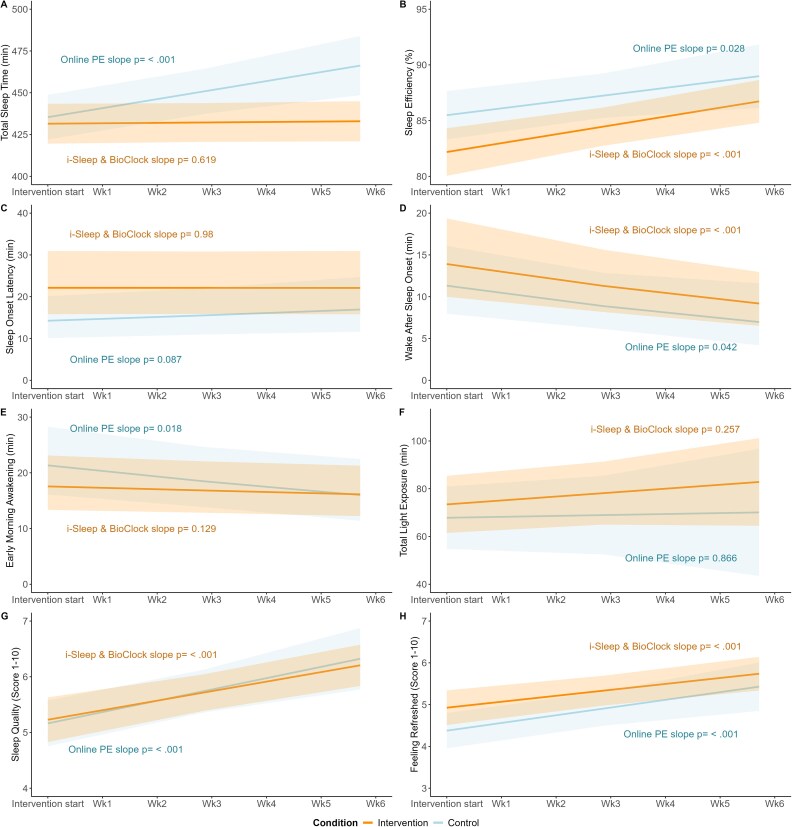
Change of sleep and light exposure diary outcomes from start to end of the intervention period. *Note.* Changes in sleep and light exposure diary variables (secondary sleep outcomes) from start to end of the intervention period with slopes for each group. Intervention group = “i-Sleep & BioClock”, Control group = Online PE. Graphs represent estimated means over time with 95% confidence intervals shown in the shaded regions.

Figure 3 displays the changes in sleep diary outcomes for intervention and control group. Total sleep time (TST) showed a significant main effect of time (*β* = 0.77, *p*<.001), indicating an increase in TST over time, with a significant time x treatment interaction in favor of the control group (*β* = -0.73, *p*<.001), see Fig. 3, Panel A. For the other sleep outcomes, there were significant within-group improvements but no significant time x treatment interaction effects. Detailed estimates for all outcomes of the sleep and light exposure diary are shown in Table S4 of the Supplementary Material.

#### Chronobiology outcomes

**Figure 4:**
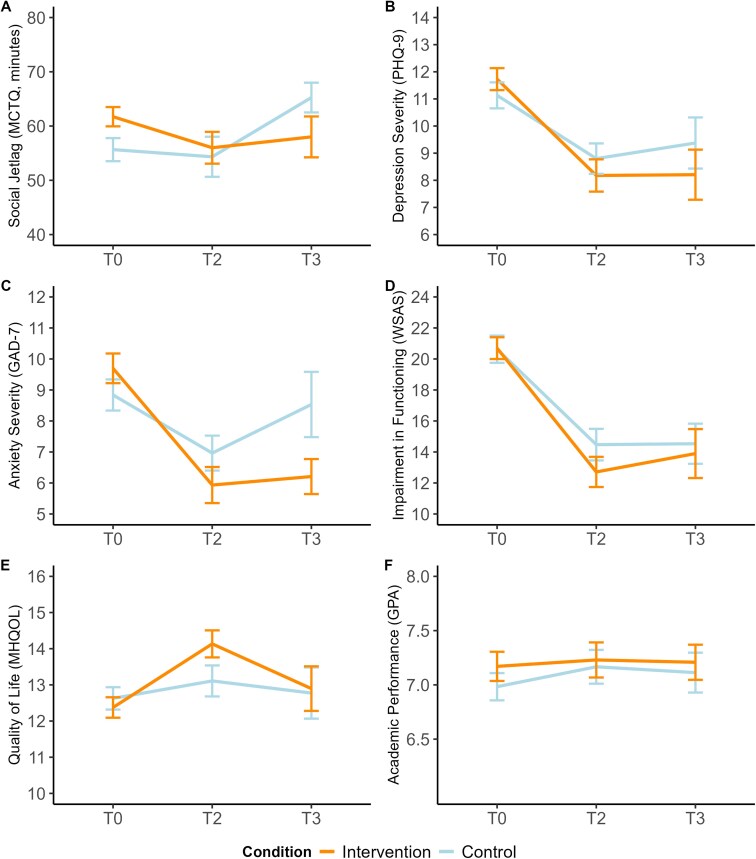
Interaction plots of effects on secondary chronobiology, mental health, and functioning outcomes. *Note.* Changes in secondary chronobiology, mental health, and functioning outcomes over time split by group. Values are based on raw (unadjusted) data. Error bars indicate standard errors. T0 = baseline, T2 = 6 weeks (post-treatment), T3 = 18 weeks (follow-up).

Social jetlag showed a no significant time x treatment effect at the primary endpoint T2 but showed a significant time x treatment interaction at follow-up, *t*(211.0) = -2.02, *p*=.04, between-group *d* = 0.36 (Fig. 4, Panel A). However, when applying Holm’s correction for multiple comparisons, this effect became non-significant at *p*=.29. Average weekly sleep duration did not show any significant main nor interaction effects.

#### Mental health and functioning outcomes

For depressive symptoms, there was no significant time x treatment interaction, *t*(199.5) = -1.36, *p*=.17, between-group *d* = -0.28, while within-group effects were large in the intervention group (*d* = -0.81) and moderate in the control group (*d* = -0.53), see Fig. 4, Panel B. Still, the within-group changes in depressive symptoms were not above the threshold (MCID) for clinical relevance in either group, with an average reduction from baseline to post-treatment of 3.5 points on the PHQ-9 in the intervention group and 2.3 points in the control group. For anxiety symptoms we also found a non-significant time x treatment interaction at T2, *t*(192.1) = -1.34, *p*=.18, between-group *d* = -0.39, but a significant interaction at T3 in favor of the intervention, *t*(195.6) = -2.35, *p*=.02, between-group *d* = -0.66 (Fig. 4, Panel C). This effect became non-significant at *p*=.16 when applying Holm’s correction. The intervention group showed a clinically relevant average reduction of 4.1 points on the GAD-7 from baseline to post-treatment, whereas the control group's 1.8-point reduction was not clinically meaningful (< 4 point change). No significant interaction effects were found in functioning while both groups improved over time (intervention *d* = -1.01; control *d* = -0.78), see Fig. 4, Panel D. For QoL we found a significant time x treatment effect favoring the intervention group at post-treatment, *t*(195.1) = 2.28, *p*=.02, between-group *d* = 0.43, but this was not maintained at follow-up, *t*(197.1) = 1.30, *p*=.20 (Fig. 4, Panel E). With Holm’s correction, the effect at T2 became non-significant at *p*=.18. Academic performance did not show any significant main nor interaction effects (Fig. 4, Panel F).

### User satisfaction and therapeutic Alliance

On average, the *“i-Sleep & BioClock”* intervention received a CSQ-8 score of 23.5 (SD 4.0) and the *“Sleep Well”* psychoeducation received a 20.6 (SD 4.0) by those students who completed T2 and who logged in at least once, which was not significantly different. The proportion of students who were highly satisfied with their assigned program (CSQ-8 score > 20) out of those who completed the T2 questionnaire was 73% (32/44) in the intervention group and 55% (27/49) in the control group.

Students in intervention group who logged in at least once (*n* = 44) rated their therapeutic alliance with the e-coach (WAI-I) at post-test with a mean score of 44.3 (SD 7.2), which is 67% of the highest achievable score on this questionnaire.

### Adverse events

No adverse events were reported at T1. A total of two events were reported at T2, one in the control group (one falling incident), and one in the intervention group (a minor incident, not specified). There was one adverse event reported at T3 in the intervention group (biking accident without injuries).

## Discussion

This study presents the effectiveness of a guided digital CBT-I intervention enriched with elements relating to the circadian rhythm (*“i-Sleep & BioClock”*) versus digital psychoeducation and sleep monitoring *(“Sleep Well”*) for university students. The aims of this study were to evaluate the intervention effects on sleep, mental health and functioning outcomes directly post-treatment, and to evaluate whether effects were maintained at follow-up. Our findings show that there were no significant time-by-treatment interaction effects on insomnia severity and most secondary outcomes, meaning that the intervention was not more effective than digital psychoeducation and sleep monitoring.

The between-group effect size we found for insomnia symptoms (*d* = -0.31; *p*=.14) at post-test was smaller than effects in prior studies of digital CBT-I interventions on sleep outcomes in university students and young adults. Several meta-analyses and RCTs report moderate to large effect sizes (ranging from *d* = 0.5 to 0.8) for guided digital CBT-I in students with insomnia [22, 23, 47, 48]. Effects were larger in those studies with passive controls or no treatment compared to meta-analyses with mixed control groups. Similar moderate to large effects have been found for fully automated or self-guided CBT-I compared to active controls or waitlist in students as well as adults with insomnia [49–51]. Studies on students usually report lower between-group effect sizes than those on the general population, where between-group effect sizes on insomnia severity and related outcomes reach *d* = 0.8 or above [18, 19, 52–56].

Several explanations for the small between-group effects on insomnia severity in our study should be considered. First, creating awareness of and monitoring sleep and light exposure patterns might be a valuable tool for university students that may lead to behavioral changes and may therefore drive the improvements in both intervention and control group. Additionally, students may respond particularly well to cognitive and psychoeducational content, given their academic background and familiarity with theoretical content. However, we expected that the additional intervention components, especially sleep restriction to increase sleep pressure, would lead to superior outcomes than psychoeducation and sleep monitoring alone. Second, the improvements observed might partly reflect non-specific effects, such as regression to the mean, natural symptom fluctuations, and participant expectations, particularly in the absence of a passive control group. Still, such non-specific factors also exist in other trials of digital CBT-I that report larger between-group effects, suggesting that the comparatively smaller effects in our study are unlikely to be explained by these factors alone. Third, adherence to the intervention was likely an issue. Only 39% of the intervention group completed the second module, in which sleep restriction was introduced. Sleep restriction is one of the core therapeutic elements in CBT-I and is considered very effective for improving sleep outcomes [57]. However, sleep restriction can be demanding, and students may require more support and receive more tailored advice to adhere to sleep restriction due to their irregular lifestyle and competing social or academic commitments. Students often face irregular externally driven schedules, making it harder to maintain a consistent sleep–wake rhythm. Prior studies have implemented strategies such as regular telephone support or video conferencing to facilitate compliance [58–60]. Although we included an intake call with the coach, more regular support might have improved adherence. Half of the students in the intervention group engaged only with the psychoeducation module (Module 1), which mirrors the content provided to the control group. Additionally, we suspect that many students did not fully adhere to the sleep restriction, which may have contributed to the similar effect sizes observed across both groups. Connected to this, it is possible that in our sample insomnia symptoms at baseline were not severe enough to motivate participants to fully engage in the interventions, which could partly explain the low adherence and high dropout percentage in both groups. Fourth, the control intervention was relatively rich in psychoeducational content and also included some advice on stimulus control and chronotype assessment. Both interventions were delivered via an engaging, professionally designed online platform, which may have supported user engagement, reducing the relative advantage of guided CBT-I.

Interestingly, the control condition also showed improvements in insomnia severity and secondary outcomes like depression and anxiety. The symptom reductions were comparable to those in the intervention group, however, on average, neither group reached clinically important improvements. Since mood and anxiety symptoms tend to wax and wane the improvements might in part be due by the passage of time. It is of course also possible that the psychoeducation itself was effective in reducing the symptoms. However, psychoeducation alone is generally considered insufficient to improve sleep in the general population [16, 61], especially in the long-term. On the other hand, it may have been beneficial for students with limited knowledge on sleep or those with very irregular schedules, which is supported by some prior studies indicate small to moderate effects of sleep hygiene education [62]. Still, psychoeducation has been found to be less effective than CBT-I [62] and prior literature suggests that core CBT-I components such as sleep restriction and cognitive restructuring, are needed to achieve robust effects [61, 63]. Yet, our control condition included more than basic sleep hygiene, incorporating elements such as stimulus control and circadian rhythm-related content, making it comparably stronger than other sleep hygiene/ psychoeducation conditions [63]. However, in designing the control intervention, we carefully restricted the content to theoretical information and students were not guided to actively change their behavior. Only three control group students opted to pursue CBT-I after completing the study, which could indicate limited additional need, although contextual factors, such as completing their studies, starting employment or experiencing other life changes may have also played a role. Ultimately, we cannot draw any conclusions on the benefits of psychoeducation alone based on this study, due to the absence of a no-treatment control condition and potential time effects.

Regarding sleep and light exposure diary outcomes, even though many outcomes improved over time, guided CBT-I did not outperform digital psychoeducation. Total sleep time (TST) had a significant interaction effect in favor of the control group. This means that students in the control group had a larger increase in hours spent sleeping than students in the intervention group over time. This might be attributable to sleep restriction in the intervention group. Sleep restriction involves spending less time in bed than usual, often leading to an initial decrease of total sleep time. However, TST typically increases over time, as it is recommended to increase the sleep duration by 15 minutes each week once the sleep is more consolidated and the weekly average sleep efficiency is sufficiently high (>85%).

Regarding secondary outcomes, there were no significant interaction effects except for an improvement in quality of life at post-treatment, and lower anxiety and social jetlag at follow-up for the intervention group. This is similar to the prior literature, showing small to moderate effects of digital CBT-I on anxiety (*d* = 0.35) [23, 64], and moderate effects on QoL (*d* = 0.47) [65]. In contrast, we did not find the same in our prior pilot study on the same intervention, in which QoL did not change significantly [66]. QoL is a broad measure that may be sensitive to small changes across multiple domains. It is possible that participants perceived benefits from the intervention, such as increased structure, greater self-efficacy, or a sense of support, which were not otherwise captured in symptom severity measures. In addition, the interaction effect of anxiety at follow-up (*d* = 0.48, *p*=.02), possibly suggesting that the intervention's effect on anxiety symptoms emerges more gradually over time. This delayed effect could be interpreted in light of cognitive-behavioral models, which propose that improvements in anxiety often follow behavioral activation, sustained practice and reinforcement of adaptive behaviors [67]. However, it is important to note that many other between-group comparisons did not reach statistical significance and that the findings in secondary outcomes might also reflect chance findings due to the large number of comparisons, as indicated by the correction for multiple comparisons.

This study has several limitations. First, our study had a high dropout rate of 48% at post-test, although this was anticipated and accounted for in the sample size calculation it still poses challenges for the interpretation of our findings. Although the sensitivity analysis indicated no major issues, the high level of dropout warrants cautious interpretation, as attrition-related biases could act in unpredictable ways, potentially leading to either overestimation or underestimation of the true effects. Second, the intervention and control condition were strictly not structurally equivalent, as the control condition received less intensive content and no coaching support. This created a challenge in isolating which specific components of the intervention account for the observed effects. Third, we did not assess possible underlying mental health conditions or comorbidities. Based on the reports on medication and psychotherapy in the baseline assessment, we can suspect that a fair number of students could be dealing with comorbid ADHD, depression and anxiety. This is important as the presence of comorbidities may have influenced participants' baseline functioning and responsiveness to the intervention, posing a potential source of confounding. A fourth limitation is that we only used self-report questionnaires and sleep and light exposure diaries. It would have been beneficial to add actigraphy for sleep and physical activity and light sensors for light exposure, to receive more objective data. Lastly, the 18-week follow-up fails to capture any long-term effect. It is possible that digital CBT-I would outperform online psychoeducation in the longer-term [68].

In summary, digital CBT-I was not superior to digital psychoeducation and sleep monitoring to improve sleep and mental well-being in the university students. Future research should identify factors for early disengagement and dropout, investigate which specific intervention components drive improvements in sleep outcomes, including the role of circadian rhythm support, and the potential of brief digital sleep interventions such as our control condition.

## Supplementary Material

20251105_Supplementary_Material_final_zsaf357

## Deposit of material in a data repository

The datasets generated and analysed during the current study are available from the corresponding author on reasonable request, and in the DataVerse repository after study completion.

## References

[1] American Psychiatric Association . Diagnostic and Statistical Manual of Mental Disorders. 5th ed. Washington DC: American Psychiatric Association; 2013.

[2] Taylor DJ, Bramoweth AD, Grieser EA, Tatum JI, Roane BM. Epidemiology of insomnia in college students: relationship with mental health, quality of life, and substance use difficulties. *Behav Ther*. 2013;44(3):339–348. 10.1016/j.beth.2012.12.001 23768662

[3] Jiang XL, Zheng XY, Yang J, et al. A systematic review of studies on the prevalence of insomnia in university students. *Public Health*. 2015;129(12):1579–1584. 10.1016/j.puhe.2015.07.030 26298588

[4] Li L, Wang Y-Y, Wang S-B, et al. Prevalence of sleep disturbances in Chinese university students: a comprehensive meta-analysis. *J Sleep Res*. 2018;27(3):e12648. 10.1111/jsr.12648 29383787

[5] Lund HG, Reider BD, Whiting AB, Prichard JR. Sleep patterns and predictors of disturbed sleep in a large population of college students. *J Adolesc Health*. 2010;46(2):124–132. 10.1016/j.jadohealth.2009.06.016 20113918

[6] Becker SP, Jarrett MA, Luebbe AM, Garner AA, Burns GL, Kofler MJ. Sleep in a large, multi-university sample of college students: sleep problem prevalence, sex differences, and mental health correlates. *Sleep Health*. 2018;4(2):174–181. 10.1016/j.sleh.2018.01.001 29555131 PMC5863586

[7] Wu TT, Zou YL, Xu KD, et al. Insomnia and multiple health outcomes: umbrella review of meta-analyses of prospective cohort studies. *Public Health*. 2023;215:66–74. 10.1016/j.puhe.2022.11.021 36645961

[8] Sivertsen B, Krokstad S, Øverland S, Mykletun A. The epidemiology of insomnia: associations with physical and mental health.: the HUNT-2 study. *J Psychosom Res*. 2009;67(2):109–116. 10.1016/j.jpsychores.2009.05.001 19616137

[9] Baglioni C, Battagliese G, Feige B, et al. Insomnia as a predictor of depression: a meta-analytic evaluation of longitudinal epidemiological studies. *J Affect Disord*. 2011;135(1):10–19. 10.1016/j.jad.2011.01.011 21300408

[10] Hertenstein E, Feige B, Gmeiner T, et al. Insomnia as a predictor of mental disorders: a systematic review and meta-analysis. *Sleep Med Rev*. 2019;43:96–105. 10.1016/j.smrv.2018.10.006 30537570

[11] Vedaa Ø, Erevik EK, Hysing M, Hayley AC, Sivertsen B. Insomnia, sleep duration and academic performance: a national survey of Norwegian college and university students. *Sleep Med X*. 2019;1:100005. 10.1016/j.sleepx.2019.100005 33870164 PMC8041108

[12] Espie CA, Pawlecki B, Waterfield D, Fitton K, Radocchia M, Luik AI. Insomnia symptoms and their association with workplace productivity: cross-sectional and pre-post intervention analyses from a large multinational manufacturing company. *Sleep Health*. 2018;4(3):307–312. 10.1016/j.sleh.2018.03.003 29776626

[13] Palagini L, Hertenstein E, Riemann D, Nissen C. Sleep, insomnia and mental health. *J Sleep Res*. 2022;31(4):e13628. 10.1111/jsr.13628 35506356

[14] Hertenstein E, Benz F, Schneider CL, Baglioni C. Insomnia—a risk factor for mental disorders. *J Sleep Res*. 2023;32(6):e13930. 10.1111/jsr.13930 37211915

[15] Irwin MR, Carrillo C, Sadeghi N, Bjurstrom MF, Breen EC, Olmstead R. Prevention of incident and recurrent major depression in older adults with insomnia: a randomized clinical trial. *JAMA Psychiatry*. 2022;79(1):33–41. 10.1001/jamapsychiatry.2021.3422 34817561 PMC8733847

[16] Riemann D, Espie CA, Altena E, et al. The European Insomnia Guideline: an update on the diagnosis and treatment of insomnia 2023. *J Sleep Res*. 2023;32(6):e14035. 10.1111/jsr.14035 38016484

[17] Simon L, Steinmetz L, Feige B, Benz F, Spiegelhalder K, Baumeister H. Comparative efficacy of onsite, digital, and other settings for cognitive behavioral therapy for insomnia: a systematic review and network meta-analysis. *Sci Rep*. 2023;13(1):1929. 10.1038/s41598-023-28853-0 36732610 PMC9894949

[18] Zachariae R, Lyby MS, Ritterband LM, O'Toole MS. Efficacy of internet-delivered cognitive-behavioral therapy for insomnia – a systematic review and meta-analysis of randomized controlled trials. *Sleep Med Rev*. 2016;30:1–10. 10.1016/j.smrv.2015.10.004 26615572

[19] Van Straten A, Van Der Zweerde T, Kleiboer A, Cuijpers P, Morin CM, Lancee J. Cognitive and behavioral therapies in the treatment of insomnia: a meta-analysis. *Sleep Med Rev*. 2018;38:3–16. 10.1016/j.smrv.2017.02.001 28392168

[20] Ho FY-Y, Chung K-F, Yeung W-F, et al. Self-help cognitive-behavioral therapy for insomnia: a meta-analysis of randomized controlled trials. *Sleep Med Rev*. 2015;19:17–28. 10.1016/j.smrv.2014.06.010 25104471

[21] Chandler L, Patel C, Lovecka L, et al. Improving university students' mental health using multi-component and single-component sleep interventions: a systematic review and meta-analysis. *Sleep Med*. 2022;100:354–363. 10.1016/j.sleep.2022.09.003 36198252

[22] Saruhanjan K, Zarski AC, Bauer T, et al. Psychological interventions to improve sleep in college students: a meta-analysis of randomized controlled trials. *J Sleep Res*. 2021;30(1):e13097. 10.1111/jsr.13097 32672865

[23] Kodsi A, Bullock B, Kennedy GA, Tirlea L. Psychological interventions to improve sleep in young adults: a systematic review and meta-analysis of randomized controlled trials. *Behav Sleep Med*. 2022;20(1):125–142. 10.1080/15402002.2021.1876062 33554644

[24] Phillips AJK, Clerx WM, O’Brien CS, et al. Irregular sleep/wake patterns are associated with poorer academic performance and delayed circadian and sleep/wake timing. *Sci Rep*. 2017;7(1):3216. 10.1038/s41598-017-03171-4 28607474 PMC5468315

[25] Crouse JJ, Carpenter JS, Song YJC, et al. Circadian rhythm sleep–wake disturbances and depression in young people: implications for prevention and early intervention. *Lancet Psychiatry*. 2021;8(9):813–823. 10.1016/S2215-0366(21)00034-1 34419186

[26] Caliandro R, Streng AA, van Kerkhof LWM, van der Horst GTJ, Chaves I. Social jetlag and related risks for human health: a timely review. *Nutrients.* 2021;13(12):4543. 10.3390/nu13124543 PMC8707256 34960096

[27] Wittmann M, Dinich J, Merrow M, Roenneberg T. Social jetlag: misalignment of biological and social time. *Chronobiol Int*. 2006;23(1-2):497–509. 10.1080/07420520500545979 16687322

[28] Roenneberg T, Allebrandt Karla V, Merrow M, Vetter C. Social jetlag and obesity. *Curr Biol*. 2012;22(10):939–943. 10.1016/j.cub.2012.03.038 22578422

[29] Leerssen J, Lakbila-Kamal O, Dekkers LMS, et al. Treating insomnia with high risk of depression using therapist-guided digital cognitive, behavioral, and circadian rhythm support interventions to prevent worsening of depressive symptoms: a randomized controlled trial. *Psychother Psychosom*. 2022;91(3):168–179. 10.1159/000520282 34872087

[30] MoodLift Platform . https://moodlift.nl/. Accessed November, 2025.

[31] Hopewell S, Chan A-W, Collins GS, et al. CONSORT 2025 statement: updated guideline for reporting randomized trials. *JAMA.* 2025;333(22):1998–2005. 10.1001/jama.2025.4347 40228499

[32] Pape LM, van Straten A, Struijs SY, Spinhoven P, Antypa N. Effectiveness of a guided digital self-help intervention to improve sleep and the biological clock in university students – study protocol for a randomized controlled trial. *Internet Interv*. 2024;37:100763. 10.1016/j.invent.2024.100763 39224668 PMC11367106

[33] Morin CM, Belleville G, Bélanger L, Ivers H. The insomnia severity index: psychometric indicators to detect insomnia cases and evaluate treatment response. *Sleep.* 2011;34(5):601–608. 10.1093/sleep/34.5.601 21532953 PMC3079939

[34] Roenneberg T, Wirz-Justice A, Merrow M. Life between clocks: daily temporal patterns of human chronotypes. *J Biol Rhythms*. 2003;18(1):80–90. 10.1177/0748730402239679 12568247

[35] Kroenke K, Spitzer RL, Williams JB. The PHQ-9: validity of a brief depression severity measure. *J Gen Intern Med*. 2001;16(9):606–613. 10.1046/j.1525-1497.2001.016009606.x 11556941 PMC1495268

[36] Spitzer RL, Kroenke K, Williams JBW, Löwe B. A brief measure for assessing generalized anxiety disorder: the GAD-7. *Arch Intern Med*. 166(10):1092–1097. 10.1001/archinte.166.10.1092 16717171

[37] Mundt JC, Marks IM, Shear MK, Greist JM. The work and social adjustment scale: a simple measure of impairment in functioning. *Br J Psychiatry*. 2002;180(5):461–464. 10.1192/bjp.180.5.461 11983645

[38] van Krugten FCW, Busschbach JJV, Versteegh MM, Hakkaart-van Roijen L, Brouwer WBF. The Mental Health Quality of Life Questionnaire (MHQoL): development and first psychometric evaluation of a new measure to assess quality of life in people with mental health problems. *Qual Life Res*. 2022;31(2):633–643. 10.1007/s11136-021-02935-w 34241821 PMC8847188

[39] Kroenke K . Enhancing the clinical utility of depression screening. *CMAJ.* 2012;184(3):281–282. 10.1503/cmaj.112004 22231681 PMC3281149

[40] Toussaint A, Hüsing P, Gumz A, et al. Sensitivity to change and minimal clinically important difference of the 7-item generalized anxiety disorder questionnaire (GAD-7). *J Affect Disord*. 2020;265:395–401. 10.1016/j.jad.2020.01.032 32090765

[41] Larsen DL, Attkinsson CC, Hargreaves WA, Nguyen TD. Asessment of the client/patient satisfaction: development of a general scale. *Eval Program Plann*. 1979;2:197–207. 10245370 10.1016/0149-7189(79)90094-6

[42] Gómez Penedo JM, Berger T, Grosse Holtforth M, et al. The Working Alliance Inventory for guided Internet interventions (WAI-I). *J Clin Psychol*. 2020;76(6):973–986. 10.1002/jclp.22823 31240727

[43] Twisk JWR . Applied Longitudinal Data Analysis for Epidemiology: A Practical Guide. 2nd ed. Cambridge: Cambridge University Press; 2013.

[44] Goulet-Pelletier J-C, Cousineau D. A review of effect sizes and their confidence intervals, part I: the Cohen’s d family. *The Quantitative Methods for Psychology*. 2018;14(4):242–265. 10.20982/tqmp.14.4.p242

[45] Grier RA, Bangor A, Kortum P, Peres SC. The system usability scale. *Proceedings of the Human Factors and Ergonomics Society Annual Meeting*. 2013;57(1):187–191. 10.1177/1541931213571042

[46] Hedeker D, Gibbons RD. Application of random-effects pattern-mixture models for missing data in longitudinal studies. 1997;2:64–78. 10.1037/1082-989X.2.1.64

[47] Freeman D, Sheaves B, Goodwin GM, et al. The effects of improving sleep on mental health (OASIS): a randomised controlled trial with mediation analysis. *Lancet Psychiatry*. 2017;4(10):749–758. 10.1016/s2215-0366(17)30328-0 28888927 PMC5614772

[48] Tsai HJ, Yang AC, Zhu JD, Hsu YY, Hsu TF, Tsai SJ. Effectiveness of digital cognitive behavioral therapy for insomnia in young people: preliminary findings from systematic review and meta-analysis. *J Pers Med*. 2022;12(3):481. 10.3390/jpm12030481 PMC8949345 35330480

[49] Chang YP, Pereira T, Salinas A, Or HY, Morales M, Le ML. Effects of an email delivered cognitive behavioral therapy for insomnia in college students. *Perspect Psychiatr Care*. 2021;57(4):1685–1692. 10.1111/ppc.12736 33547638

[50] Morris J, Firkins A, Millings A, Mohr C, Redford P, Rowe A. Internet-delivered cognitive behavior therapy for anxiety and insomnia in a higher education context. *Anxiety, Stress, & Coping*. 2016;29(4):415–431. 10.1080/10615806.2015.1058924 26079158

[51] Trockel M, Manber R, Chang V, Thurston A, Taylor CB. An e-mail delivered CBT for sleep-health program for college students: effects on sleep quality and depression symptoms. *J Clin Sleep Med*. 2011;7(3):276–281. 10.5664/jcsm.1072 21677898 PMC3113967

[52] Christensen H, Batterham PJ, Gosling JA, et al. Effectiveness of an online insomnia program (SHUTi) for prevention of depressive episodes (the GoodNight study): a randomised controlled trial. *Lancet Psychiatry*. 2016;3(4):333–341. 10.1016/s2215-0366(15)00536-2 26827250

[53] Espie CA, Emsley R, Kyle SD, et al. Effect of digital cognitive behavioral therapy for insomnia on health, psychological well-being, and sleep-related quality of life: a randomized clinical trial. *JAMA Psychiatry*. 2019;76(1):21. 10.1001/jamapsychiatry.2018.2745 30264137 PMC6583463

[54] Ritterband LM, Thorndike FP, Ingersoll KS, et al. Effect of a web-based cognitive behavior therapy for insomnia intervention with 1-year follow-up: a randomized clinical trial. *JAMA Psychiatry*. 2017;74(1):68–75. 10.1001/jamapsychiatry.2016.3249 27902836

[55] Horsch CH, Lancee J, Griffioen-Both F, et al. Mobile phone-delivered cognitive behavioral therapy for insomnia: a randomized waitlist controlled trial. *J Med Internet Res*. 2017;19(4):e70. 10.2196/jmir.6524 28400355 PMC5405291

[56] Vedaa Ø, Kallestad H, Scott J, et al. Effects of digital cognitive behavioural therapy for insomnia on insomnia severity: a large-scale randomised controlled trial. *The Lancet Digital Health*. 2020;2(8):e397–e406. 10.1016/s2589-7500(20)30135-7 33328044

[57] Maurer LF, Schneider J, Miller CB, Espie CA, Kyle SD. The clinical effects of sleep restriction therapy for insomnia: a meta-analysis of randomised controlled trials. *Sleep Med Rev*. 2021;58:101493. 10.1016/j.smrv.2021.101493 33984745

[58] Tadros M, Li S, Corkish B, Upton E, Newby J, Werner-Seidler A. Cognitive behavior therapy for insomnia in university students delivered via videoconferencing groups: a pilot study. *Behav Sleep Med*. 2024;22(6):843–856. 10.1080/15402002.2024.2374258 38949071

[59] Ho FY-Y, Chung K-F, Yeung W-F, Ng TH-Y, Cheng SK-W. Weekly brief phone support in self-help cognitive behavioral therapy for insomnia disorder: relevance to adherence and efficacy. *Behav Res Ther*. 2014;63:147–156. 10.1016/j.brat.2014.10.002 25461790

[60] Jansson-Fröjmark M, Sunnhed R. Smartphone application-delivered cognitive behavioural therapy for insomnia with telephone support for insomnia disorder compared to a waitlist control: a randomised clinical trial. *J Sleep Res*. 2024;34(3):e14363. 10.1111/jsr.14363 39377371 PMC12069742

[61] Steinmetz L, Simon L, Feige B, et al. Network meta-analysis examining efficacy of components of cognitive behavioural therapy for insomnia. *Clin Psychol Rev*. 2024;114:102507. 10.1016/j.cpr.2024.102507 39504928

[62] Chung K-F, Lee C-T, Yeung W-F, Chan M-S, Chung EW-Y, Lin W-L. Sleep hygiene education as a treatment of insomnia: a systematic review and meta-analysis. *Fam Pract*. 2018;35(4):365–375. 10.1093/fampra/cmx122 29194467

[63] Furukawa Y, Sakata M, Yamamoto R, et al. Components and delivery formats of cognitive behavioral therapy for chronic insomnia in adults: a systematic review and component network meta-analysis. *JAMA Psychiatry*. 2024;81(4):357–365. 10.1001/jamapsychiatry.2023.5060 38231522 PMC10794978

[64] Ye Y-Y, Zhang Y-F, Chen J, et al. Internet-based cognitive behavioral therapy for insomnia (ICBT-i) improves comorbid anxiety and depression—a meta-analysis of randomized controlled trials. *PloS One*. 2015;10(11):e0142258. 10.1371/journal.pone.0142258 26581107 PMC4651423

[65] Alimoradi Z, Jafari E, Brostrom A, et al. Effects of cognitive behavioral therapy for insomnia (CBT-I) on quality of life: a systematic review and meta-analysis. *Sleep Med Rev*. 2022;64:101646. 10.1016/j.smrv.2022.101646 35653951

[66] Pape LM, Antypa N, Spinhoven P, et al. Open pilot study of a guided digital self-help intervention targeting sleep and the biological clock in university students using a pre-test post-test design. *Sci Rep*. 2025;15(1):21837. 10.1038/s41598-025-04891-8 40593043 PMC12218973

[67] van Dis EAM, van Veen SC, Hagenaars MA, et al. Long-term outcomes of cognitive behavioral therapy for anxiety-related disorders: a systematic review and meta-analysis. *JAMA Psychiatry*. 2020;77(3):265–273. 10.1001/jamapsychiatry.2019.3986 31758858 PMC6902232

[68] Jernelov S, Blom K, Hentati Isacsson N, et al. Very long-term outcome of cognitive behavioral therapy for insomnia: one- and ten-year follow-up of a randomized controlled trial. *Cogn Behav Ther*. 2022;51(1):72–88. 10.1080/16506073.2021.2009019 35099359

