# Supplementary material for: Effects of a guided digital intervention on sleep and mental health outcomes in university students – a randomized controlled trial
Source: Sleep. 2025 Nov 25;49(5):zsaf357. doi: 10.1093/sleep/zsaf357 (PMC13163168; doi:10.1093/sleep/zsaf357)
Supplement: 20251105_Supplementary_Material_final_zsaf357 [file 20251105_supplementary_material_final_zsaf357.docx]

**Supplementary Material**

**Title**

Effects of a Guided Digital Intervention on Sleep and Mental Health Outcomes in University Students – A Randomized Controlled Trial

**Authors**

Laura M. Pape ^1^, Annemieke van Straten ^2, 3^, Sascha Y. Struijs ^2^, Julian D. Karch ^4^, Philip Spinhoven ^1^, Niki Antypa ^1^, Caring Universities Consortium*

**Affiliations**

^1^ *Department of Clinical Psychology, Leiden University, the Netherlands* ^2^ *Department of Clinical, Neuro- and Developmental Psychology, VU University Amsterdam, the Netherlands*
^3^ *Amsterdam Public Health Research Institute, Vrije Universiteit Amsterdam, the Netherlands 
4 Department of Methodology and Statistics, Leiden University, the Netherlands*

**Corresponding author**

Niki Antypa

Email: [nantypa@fsw.leidenuniv.nl](mailto:nantypa@fsw.leidenuniv.nl)

**Table S1:** Reasons for Dropout

| **Dropout Reasons (N= 17)** | **Intervention**  **Group**  **n= 11** | **Control Group**  **n=6** |
| --- | --- | --- |
|  |  |  |
| **Personal reasons** | n | n |
| No time | 7 | 2 |
| Lost interest/ Motivation | 6 | 1 |
| Symptoms improved | 1 | 2 |
| Stressful life event | 1 | 2 |
| Found other source of help | 1 | - |
|  |  |  |
| **Intervention-related** |  |  |
| Intervention is boring | 3 | 0 |
| Technical difficulties | ⁻ | ⁻ |
| Platform difficult to navigate | - | 1 |
| Intervention too demanding | 2 | - |
| Intervention too complicated | 1 | ⁻ |
| Not what I need | 4 | 1 |
| Problems with internet connection | 1 | ⁻ |
| Uncomfortable with the degree of anonymity | 1 | ⁻ |
| Prefer face-to-face help | 2 | 2 |
|  |  |  |
| **Coach-related** |  |  |
| No support by coach | 1 | 1 |
| Different goals than coach | 2 | ⁻ |

**Table S2:** Means and effect sizes of primary and secondary outcomes

|  |  | **i-Sleep & BioClock** | | |  | **Online PE** | | |  | **Between-group** |
| --- | --- | --- | --- | --- | --- | --- | --- | --- | --- | --- |
|  |  | ***n*** | **Mean (SD)** | ***d**** |  | ***n*** | **Mean (SD)** | ***d**** |  | **Cohen's *d*** |
| **Insomnia Severity (ISI), score** | | | | |  |  |  |  |  |  |
|  | Baseline (T0) | 96 | 16.8 (4.0) |  |  | 99 | 16.5 (3.6) |  |  |  |
|  | Week 3 (T1) | 29 | 11.2 (4.1) | -1.47 |  | 27 | 12.2 (5.1) | -1.13 |  | -0.34 |
|  | Week 6 (T2) | 45 | 10.2 (3.9) | -1.72 |  | 56 | 11.1 (4.2) | -1.42 |  | -0.31 |
|  | Week 18 (T3) | 31 | 10.6 (3.9) | -1.63 |  | 33 | 10.7 (4.5) | -1.54 |  | -0.10 |
| **Depression (PHQ-9), score** | | | | |  |  |  |  |  |  |
|  | Baseline (T0) | 96 | 11.7 (4.0) |  |  | 99 | 11.1 (4.8) |  |  |  |
|  | Week 6 (T2) | 45 | 8.2 (4.0) | -0.81 |  | 55 | 8.8 (4.2) | -0.53 |  | -0.28 |
|  | Week 18 (T3) | 29 | 8.2 (5.0) | -0.80 |  | 32 | 9.4 (5.3) | -0.40 |  | -0.40 |
| **Anxiety (GAD-7), score** | | | | |  |  |  |  |  |  |
|  | Baseline (T0) | 96 | 9.7 (4.7) |  |  | 99 | 8.8 (5.0) |  |  |  |
|  | Week 6 (T2) | 45 | 5.9 (3.9) | -0.78 |  | 55 | 7.0 (4.2) | -0.38 |  | -0.39 |
|  | Week 18 (T3) | 29 | 6.2 (3.0) | -0.72 |  | 32 | 8.5 (6.0) | -0.06 |  | **-0.66**** |
| **Functioning (WSAS), score** | | | | |  |  |  |  |  |  |
|  | Baseline (T0) | 96 | 20.7 (6.9) |  |  | 99 | 20.6 (8.7) |  |  |  |
|  | Week 6 (T2) | 45 | 12.7 (6.5) | -1.01 |  | 55 | 14.5 (7.5) | -0.78 |  | -0.23 |
|  | Week 18 (T3) | 29 | 14.5 (7.3) | -0.86 |  | 32 | 13.9 (8.5) | -0.77 |  | -0.09 |
| **Quality of life (MHQoL), score** | | | | |  |  |  |  |  |  |
|  | Baseline (T0) | 96 | 12.4 (2.8) |  |  | 99 | 12.6 (3.1) |  |  |  |
|  | Week 6 (T2) | 45 | 14.1 (2.5) | 0.60 |  | 55 | 13.1 (3.2) | 0.16 |  | **0.43**** |
|  | Week 18 (T3) | 29 | 12.9 (3.3) | 0.18 |  | 31 | 12.8 (3.9) | 0.05 |  | 0.13 |
| **Academic Performance, GPA past semester** | | | | |  |  |  |  |  |  |
|  | Baseline (T0) | 85 | 7.2 (1.2) |  |  | 90 | 7.0 (1.2) |  |  |  |
|  | Week 6 (T2) | 37 | 7.2 (1.0) | 0.05 |  | 51 | 7.2 (1.1) | 0.15 |  | -0.10 |
|  | Week 18 (T3) | 24 | 7.2 (0.8) | 0.03 |  | 31 | 7.1 (1.0) | 0.11 |  | -0.08 |
| **Social Jetlag (MCTQ), hh:mm** | | | | |  |  |  |  |  |  |
|  | Baseline (T0) | 96 | 01:07 (00:51) |  |  | 99 | 00:59(00:55) |  |  |  |
|  | Week 6 (T2) | 45 | 00:51 (00:45) | -0.29 |  | 55 | 01:03 (01:01) | 0.08 |  | -0.37 |
|  | Week 18 (T3) | 30 | 00:51 (00:37) | -0.30 |  | 32 | 01:02 (00:36) | 0.05 |  | **0.36**** |
| **Average Weekly Sleep Duration (MCTQ), hh:mm** | | | | | | | |  |  |  |
|  | Baseline (T0) | 96 | 07:09 (01:36) |  |  | 99 | 07:36 (01:45) |  |  |  |
|  | Week 6 (T2) | 45 | 07:32 (01:12) | 0.23 |  | 55 | 07:46 (01:29) | 0.10 |  | 0.12 |
|  | Week 18 (T3) | 30 | 07:31 (01:26) | 0.22 |  | 32 | 07:58 (01:33) | 0.21 |  | 0.00 |
|  |  |  |  |  |  |  |  |  |  |  |
| *Note*. *d** = Within-group Cohen's *d*. Between-group Cohen’s *d* = difference in differences. ** Significant time x treatment effect (p<.05). | | | | | | | | | | |

**Table S3**: Effects of guided digital CBT-I versus online PE on primary and secondary outcomes

|  |  |  |  |  |  |
| --- | --- | --- | --- | --- | --- |
| **Linear Mixed Model Effects** | | | | | |
| **Outcome** | | **Estimate** | **SE** | ***t-value*** | ***p-value*** |
| **Insomnia Severity (ISI), score** | |  |  |  |  |
|  | Intercept | 16.76 | 0.41 | 40.97 | <.001** |
|  | Treatment (CBT-I) | 0.26 | 0.57 | 0.45 | 0.66 |
|  | Time (T2) | -6.39 | 0.58 | -11.02 | <.001** |
|  | Time (T2) X Treatment | -1.17 | 0.79 | -1.49 | 0.14 |
|  | Time (T3) | -6.09 | 0.67 | -9.06 | <.001** |
|  | Time (T3) X Treatment | 0.49 | 0.94 | 0.52 | 0.60 |
| **Depression (PHQ-9), score** | |  |  |  |  |
|  | Intercept | 11.13 | 0.44 | 25.00 | <.001* |
|  | Treatment (CBT-I) | 0.60 | 0.63 | 0.94 | 0.35 |
|  | Time (T2) | -2.24 | 0.54 | -4.10 | <.001** |
|  | Time (T2) X Treatment | -1.10 | 0.81 | -1.36 | 0.17 |
|  | Time (T3) | -1.79 | 0.68 | -2.64 | 0.01* |
|  | Time (T3) X Treatment | -1.57 | 0.98 | -1.60 | 0.11 |
| **Anxiety (GAD-7), score** | |  |  |  |  |
|  | Intercept | 8.84 | 0.47 | 18.91 | <.001** |
|  | Treatment (CBT-I) | 0.86 | 0.67 | 1.29 | 0.19 |
|  | Time (T2) | -2.08 | 0.57 | -3.63 | <.001** |
|  | Time (T2) X Treatment | -1.14 | 0.85 | -1.34 | 0.18 |
|  | Time (T3) | -0.29 | 0.71 | -0.41 | 0.68 |
|  | Time (T3) X Treatment | -2.42 | 1.03 | -2.35 | **0.02*** |
| **Functioning (WSAS), score** | |  |  |  |  |
|  | Intercept | 20.63 | 0.78 | 26.56 | <.001** |
|  | Treatment (CBT-I) | 0.07 | 1.11 | 0.07 | 0.94 |
|  | Time (T2) | -6.08 | 1.00 | -6.08 | <.001** |
|  | Time (T2) X Treatment | -1.57 | 1.48 | -1.06 | 0.29 |
|  | Time (T3) | -5.68 | 1.24 | -4.57 | <.001** |
|  | Time (T3) X Treatment | -0.92 | 1.80 | -0.51 | 0.61 |
| **Quality of life (MHQoL), score** | |  |  |  |  |
|  | Intercept | 12.62 | 0.30 | 41.63 | <.001** |
|  | Treatment (CBT-I) | -0.25 | 0.43 | -0.58 | 0.56 |
|  | Time (T2) | 0.43 | 0.35 | 1.26 | 0.21 |
|  | Time (T2) X Treatment | 1.17 | 0.51 | 2.28 | **0.02*** |
|  | Time (T3) | -0.16 | 0.44 | -0.37 | 0.71 |
|  | Time (T3) X Treatment | 0.82 | 0.63 | 1.30 | 0.20 |
| ***Table continued*** | | | | | |
| **Linear Mixed Model Effects** | | | | | |
|  | **Outcome** | **Estimate** | **SE** | ***t-value*** | ***p-value*** |
| **Academic Performance, GPA past semester** | | | | | |
|  | Intercept | 6.99 | 0.12 | 58.19 | <.001** |
|  | Treatment (CBT-I) | 0.18 | 0.17 | 1.06 | 0.29 |
|  | Time (T2) | 0.07 | 0.11 | 0.62 | 0.54 |
|  | Time (T2) X Treatment | -0.13 | 0.16 | -0.82 | 0.42 |
|  | Time (T3) | 0.16 | 0.13 | 1.25 | 0.21 |
|  | Time (T3) X Treatment | -0.18 | 0.20 | -0.94 | 0.35 |
| **Social Jetlag (MCTQ), minutes** | |  |  |  |  |
|  | Intercept | 59.52 | 5.19 | 17.42 | <.001** |
|  | Treatment (CBT-I) | 7.90 | 7.39 | 1.38 | 0.17 |
|  | Time (T2) | 3.16 | 6.79 | 0.15 | 0.88 |
|  | Time (T2) X Treatment | -20.62 | 10.05 | -1.94 | 0.05 |
|  | Time (T3) | -3.19 | 8.44 | 0.69 | 0.49 |
|  | Time (T3) X Treatment | -15.96 | 12.14 | -2.02 | **0.04*** |
| **Average Weekly Sleep Duration (MCTQ), minutes** | | | | | |
|  | Intercept | 456.03 | 9.57 | 47.63 | <.001** |
|  | Treatment (CBT-I) | -27.07 | 12.31 | -1.98 | 0.05 |
|  | Time (T2) | 2.31 | 12.31 | 0.19 | 0.85 |
|  | Time (T2) X Treatment | 16.80 | 18.22 | 0.92 | 0.36 |
|  | Time (T3) | 16.26 | 15.30 | 1.06 | 0.29 |
|  | Time (T3) X Treatment | 4.79 | 22.01 | 0.22 | 0.83 |
|  |  |  |  |  |  |
| *Note*. Academic performance (GPA), Social Jetlag (MCTQ) and Average Weekly Sleep Duration (MCTQ) showed violations in assumptions (residuals were skewed). Estimates of square root transformed analysis are reported for Social Jetlag. Estimates of Robust LMM analysis are reported for GPA and Average Sleep Duration. | | | | | |

**Table S4**: Effects of guided digital CBT-I versus online PE on sleep and light exposure diary outcomes

|  |  |  |  |  |  |  |  |  |  |  |  |  |  |  |  |
| --- | --- | --- | --- | --- | --- | --- | --- | --- | --- | --- | --- | --- | --- | --- | --- |
|  |  | **Linear Mixed Model** | | | |  | **Negative Binominal Mixed Model** | | | |  | **Logistic Binominal Mixed Model** | | | |
| **Outcome** | | **Estimate** | **SE** | ***t-value*** | ***p-value*** |  | **Estimate** | **95% CI** | ***Z-value*** | ***p-value*** |  | **Estimate** | **SE** | ***Z-value*** | ***p-value*** |
| **Total Sleep Time (minutes)** | | |  |  |  |  |  |  |  |  |  |  |  |  |  |
|  | Intercept | 435.4 | 6.81 | 63.92 | <.001** |  |  |  |  |  |  |  |  |  |  |
|  | Treatment (CBT-I) | -3.91 | 9.13 | -0.43 | 0.66 |  |  |  |  |  |  |  |  |  |  |
|  | Time | 0.77 | 0.2 | 3.79 | <.001** |  |  |  |  |  |  |  |  |  |  |
|  | Time X Treatment | -0.73 | 0.21 | -3.4 | **<.001**** |  |  |  |  |  |  |  |  |  |  |
| **Sleep Efficiency (%)** | |  |  |  |  |  |  |  |  |  |  |  |  |  |  |
|  | Intercept | 0.87 | 0.28 | 31.62 | <.001** |  |  |  |  |  |  |  |  |  |  |
|  | Treatment (CBT-I) | -0.39 | 0.31 | -2.04 | **0.04*** |  |  |  |  |  |  |  |  |  |  |
|  | Time | 0.12 | 0.09 | 2.21 | **0.03*** |  |  |  |  |  |  |  |  |  |  |
|  | Time X Treatment | 0.07 | 0.1 | 0.42 | 0.67 |  |  |  |  |  |  |  |  |  |  |
| **Sleep Quality (score)** | |  |  |  |  |  |  |  |  |  |  |  |  |  |  |
|  | Intercept | 5.61 | 0.21 | 22.66 | <.001** |  |  |  |  |  |  |  |  |  |  |
|  | Treatment (CBT-I) | 0.07 | 0.29 | 0.22 | 0.82 |  |  |  |  |  |  |  |  |  |  |
|  | Time | 0.03 | 0.01 | 3.82 | **<.001**** |  |  |  |  |  |  |  |  |  |  |
|  | Time X Treatment | -0.01 | 0.01 | -0.5 | 0.61 |  |  |  |  |  |  |  |  |  |  |
| **Refreshed in the morning (score)** | | |  |  |  |  |  |  |  |  |  |  |  |  |  |
|  | Intercept | 4.37 | 0.22 | 20.34 | <.001** |  |  |  |  |  |  |  |  |  |  |
|  | Treatment (CBT-I) | 0.54 | 0.3 | 1.82 | 0.07 |  |  |  |  |  |  |  |  |  |  |
|  | Time | 0.03 | 0.01 | 3.47 | **<.001**** |  |  |  |  |  |  |  |  |  |  |
|  | Time X Treatment | -0.01 | 0.01 | -0.66 | 0.51 |  |  |  |  |  |  |  |  |  |  |
| **Total light exposure (minutes)** | | |  |  |  |  |  |  |  |  |  |  |  |  |  |
|  | Intercept | 42.19 | 0.17 | 15.80 | <.001** |  |  |  |  |  |  |  |  |  |  |
|  | Treatment (CBT-I) | 0.18 | 0.32 | 0.76 | 0.44 |  |  |  |  |  |  |  |  |  |  |
|  | Time | -2.71 | 0.00 | -0.32 | 0.75 |  |  |  |  |  |  |  |  |  |  |
|  | Time X Treatment | 0.00 | 0.00 | 0.84 | 0.40 |  |  |  |  |  |  |  |  |  |  |
|  |  |  |  |  |  |  |  |  |  |  |  |  |  |  |  |
| ***Table continued*** | |  |  |  |  |  |  |  |  |  |  |  |  |  |  |
|  |  | **Linear Mixed Model** | | | |  | **Negative Binominal Mixed Model** | | | |  | **Logistic Binominal Mixed Model** | | | |
| **Outcome** | | **Estimate** | **SE** | ***t-value*** | ***p-value*** |  | **Estimate** | **95% CI** | ***Z-value*** | ***p-value*** |  | **Estimate** | **SE** | ***Z-value*** | ***p-value*** |
| **Sleep Onset Latency (minutes)** | | |  |  |  |  |  |  |  |  |  |  |  |  |  |
|  | Intercept |  |  |  |  |  | 1.19 | (0.91, 1.58) | 1.20 | 0.23 |  |  |  |  |  |
|  | Treatment (CBT-I) |  |  |  |  |  | 1.53 | (1.05, 2.22) | 2.37 | **0.02*** |  |  |  |  |  |
|  | Time |  |  |  |  |  | 1.00 | (0.99, 1.01) | 0.40 | 0.69 |  |  |  |  |  |
|  | Time X Treatment |  |  |  |  |  | 1.00 | (0.99, 1.00) | -0.96 | 0.34 |  |  |  |  |  |
| **Wake After Sleep Onset (minutes)** | | |  |  |  |  |  |  |  |  |  |  |  |  |  |
|  | Intercept |  |  |  |  |  | 0.91 | (0.68, 1.22) | -0.60 | 0.55 |  |  |  |  |  |
|  | Treatment (CBT-I) |  |  |  |  |  | 1.19 | (0.81,1.75) | 0.88 | 0.38 |  |  |  |  |  |
|  | Time |  |  |  |  |  | 0.99 | (0.98, 1.00) | -2.15 | **0.03*** |  |  |  |  |  |
|  | Time X Treatment |  |  |  |  |  | 1.00 | (0.99, 1.02) | 0.58 | 0.56 |  |  |  |  |  |
| **Early Morning Awakening (minutes)** | | | |  |  |  |  |  |  |  |  |  |  |  |  |
|  | Intercept |  |  |  |  |  | 1.59 | (1.26, 2.01) | 4.03 | <.001** |  |  |  |  |  |
|  | Treatment (CBT-I) |  |  |  |  |  | 0.88 | (0.64, 1.21) | -0.77 | 0.44 |  |  |  |  |  |
|  | Time |  |  |  |  |  | 0.99 | (0.98, 1.00) | -2.71 | **0.01*** |  |  |  |  |  |
|  | Time X Treatment |  |  |  |  |  | 1.00 | (0.99, 1.01) | 1.55 | 0.12 |  |  |  |  |  |
| **Screen time (yes/no)** | |  |  |  |  |  |  |  |  |  |  |  |  |  |  |
|  | Intercept |  |  |  |  |  |  |  |  |  |  | 2.99 | 0.37 | 7.94 | <.001** |
|  | Treatment (CBT-I) |  |  |  |  |  |  |  |  |  |  | -1.22 | 0.45 | -2.17 | **0.01*** |
|  | Time |  |  |  |  |  |  |  |  |  |  | -0.01 | 0.01 | -0.63 | 0.52 |
|  | Time X Treatment |  |  |  |  |  |  |  |  |  |  | 0.01 | 0.01 | 1.07 | 0.29 |
|  |  |  |  |  |  |  |  |  |  |  |  |  |  |  |  |
| *Note*. The unit for the outcome variable SOL and WASO were 15 minutes intervals recoded into levels. Estimates and 95% CI’s are calculated for the exponentiated results. | | | | | | | | | | | | | | | |

**List of Captions for Supplementary Tables**

**Table S1:** Reasons for Dropout

**Table S2:** Means and effect sizes of primary and secondary outcomes

**Table S3**: Effects of guided digital CBT-I versus online PE on primary and secondary outcomes

**Table S4**: Effects of guided digital CBT-I versus online PE on sleep and light exposure diary outcomes
